# Hongfu Chu: World renowned entomologist

**DOI:** 10.1007/s13238-016-0339-5

**Published:** 2016-11-04

**Authors:** Dayong Xue, Hongxiang Han

**Affiliations:** Institute of Zoology, Chinese Academy of Sciences, Beijing, 100101 China

Professor Hongfu Chu (Hongfu Zhu) is a renowned Chinese biologist, entomologist and taxonomist who specializes in research fields such as insect immature, insect morphology, insect taxonomy and plant protection. He is a pioneer of taxonomist and the founder of Lepidoptera and aphid systematics. Hungfu Chu is the first person to erect the nomenclature of the chaetotaxy of lepidopteran larvae, and he introduced numerical taxonomy and cladistics to China. He also compiled the textbook *Theoretical Fundamentals of Animal Systematics*, which is the first theoretical monograph in animal systematics in China. Hongfu Chu devoted all of his time to the study of entomology, and he made magnificent contributions in establishing and developing entomology and insect taxonomy in China.

Born in 1910, Hongfu Chu received traditional education in his hometown, Nantong, Jiangsu province. He received a BS degree from the Department of Biology at Tsinghua University in 1935, and afterward, worked as an assistant professor in Tsinghua University. During his study at Tsinghua University, he showed great interests in zoology, and especially, entomology. In 1941, he was sent to Illinois University to study entomology and ecology under entomologist Prof. W. P. Hayes and ecologist V. E. Shelford. After receiving his Master’s Degree in 1942, Hongfu Chu continued his graduate studies and received a Ph.D. degree in 1945. After graduation, Hongfu Chu accepted an offer from the Natural Museum of Illinois, where he researched the taxonomy of sawfly under Prof. H. H. Ross from 1945 to 1946. Shortly after his time at the Natural Museum of Illinois, Hongfu Chu worked as a guest professor at Wesleyan University from 1946 to 1947. In 1947, due to his commitment to the development of entomology in China, Hongfu Chu returned to China with his family.

**Figure 1:**
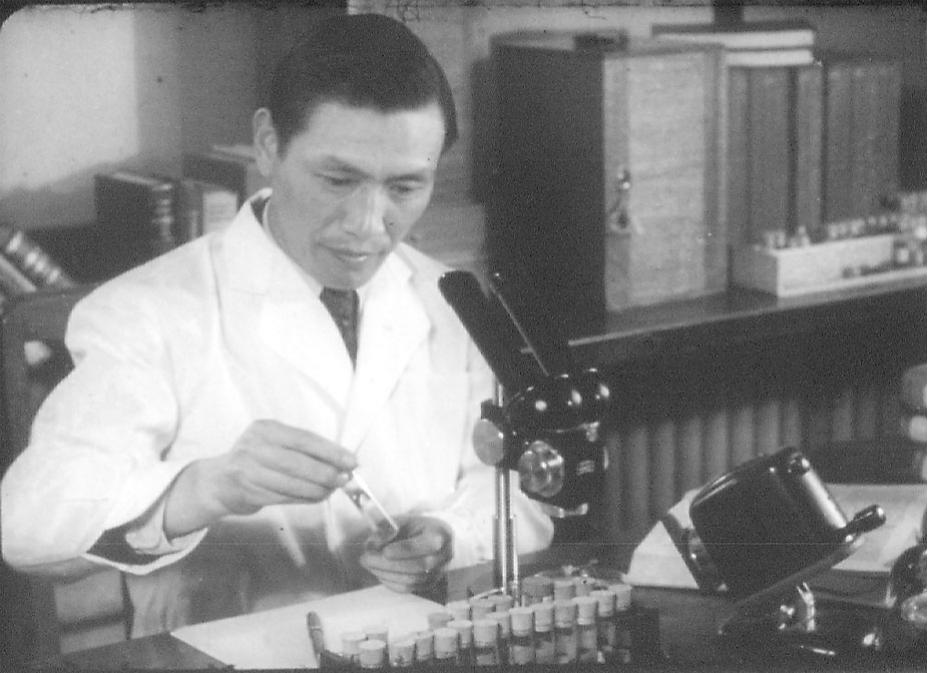
Prof. Hongfu Chu. Working in the laboratory (mid-1950s)

In 1950, Hongfu Chu accepted commission from President Moruo Guo of the Chinese Academy of Science to initiate the establishment of the Institute of Entomology (which later became a major part of the Institute of Zoology, Chinese Academy of Sciences, Beijing). In addition to insect taxonomy and insect morphology, Hongfu Chu added resources on entomology, insect histology, insect physiology, insect ecology, insect toxicology and pesticide, and invited and hired many famous entomologists to join the institute, such as entomologist Chongle Liu, Banghua Cai, insect morphologist Jinren Lu, insect physiologist Junde Qin, and insect ecologist Shijun Ma. After three years of great efforts, the Institute of Entomology was established, including the first department of insect toxicology and pesticide in the world (Fig. 1).

During his professional career, Hongfu Chu had held the positions of Vice Director and Acting Director of Institute of Entomology and Institute of Zoology, Chinese Academy of Sciences; Head of Agricultural Division of the National Science Commission; Member of Academic Committee of the Chinese Academy of Agricultural Sciences; Head of the Board of Directors of the Entomological Society of China; Chairman of the XIX International Congress of Entomology; Vice Chairman and Chairman of Board of Editors of *Fauna Sinica* and *Economic Insect Fauna of China*; Chief Editor or Editor of *Acta Entomologica Sinica*, *Chinese Journal of Entomology*, *Acta Zootaxonomica Sinica*, *Sinozoologica*, *Discovery and Innovation* and *Annals of Entomology*.

Before 1970, Hongfu Chu engaged in research regarding two main areas: (i) insect larvae, life history, and biology, and (ii) forecasting, prediction and control of pests. Later, he mainly engaged in researches of insect taxonomy.

Hongfu Chu initiated studies on the morphology of immature insects. In 1949, Chu and Cutkomp published *How to Know the Immature Insect*, which has been used as a textbook and reference book by students of entomology in America. The morphological characters of immature insects of 24 orders were described, and illustrations of larval morphological characters, and keys to 16 representative families, were provided. From then on, research on moth larvae in China greatly increased. Hongfu Chu erected the nomenclature of the chaetotaxy of lepidopteran larvae in 1956, thus prompting important publications such as *Economic Insect Fauna of China* (*Fasc. 7, Lepidoptera Noctuidae III larvae*) and *Iconography of Larvae of Moths*.

During most of his life, Hongfu Chu worked mostly on important agricultural pests, especially cotton pests, and studied the identification, forecasting, prediction and control of said pests. He published several articles in these fields, such as *An Introduction to Aphidology* and *Cotton Pests in China*. Hongfu Chu’s most significant contribution was that he identified many serious pests such as *Sitodiplosis mosellana* (Ghin) and wheat sawfly *Dolerus tritici*. He also greatly improved the development of plant protection in China.

Hongfu Chu mainly focused his research on the taxonomy of Lepidoptera in China from 1950s to his later days. He established the Lepidoptera workgroup and educated tens of graduated students. He also organized the monumental work of *Iconocraphia Heterocerorum Sinicorum* I–IV, which was the milestone of moth taxonomy in China. Hongfu Chu described 7 new genera, 159 new species, and 21 new subspecies throughout his taxonomic career. All new taxa described by him, except the wheat sawfly *Dolerus tritici* Chu, were Lepidoptera, including Agaristidae, Saturniidae, Hepialidae, Bombycidae, Brahmaeidae, Drepanidae, Noctuidae, Epicopeiidae, Geometridae, Sphingidae and Thyrididae. Hongfu Chu had led the editing of the huge series *Fauna Sinica* and *Economic Insect Fauna of China*. The latter series of monograph was awarded the Nature Science Prize by the Chinese Academy of Sciences (2000) and National Science Prize (2001).

**Figure 2:**
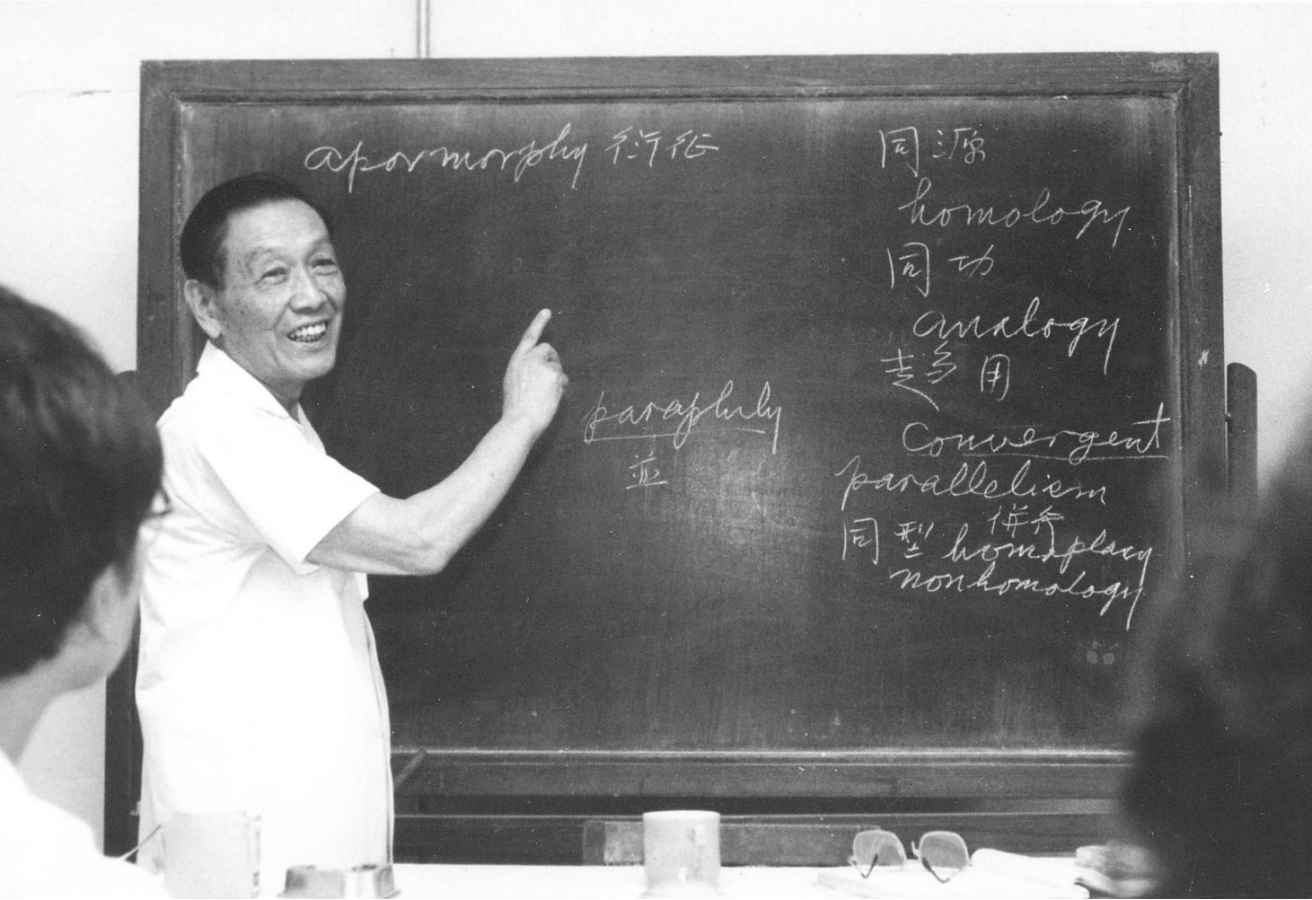
Hongfu Chu. Lecturing on cladistics in the Institute of Zoology, Chinese Academy of Sciences, Beijing, China (1984)

To facilitate the development of entomology in China, Hongfu Chu placed great emphasis on inducing important reference books, new theories, and methods. He presided over the translation of a series of the most important monographs in zoology and entomology, such as: *International Code of Zoological Nomenclature* (the Second and Third Editions), *English-Chinese Dictionary of Entomology* (the First and Second Editions). Hongfu Chu introduced numerical taxonomic methods to China in 1975, and he began to study and teach phylogenetic systematics in early 1980s, and published *Theoretical Fundamentals of Animal Systematics* in 1987, which was the first monograph on this subject in China (Fig. 2).

Hongfu Chu often told students, researchers, and assistants, “knowledge is infinite, and one need cherish the time.” He was known to transfer the projects which had preliminary achievements to young students, and he never monopolized the outcome. He is the epitome of the old-generation of Chinese scientists, those who devote his or her whole life to science and country.
